# Phenotyping Local Eggplant Varieties: Commitment to Biodiversity and Nutritional Quality Preservation

**DOI:** 10.3389/fpls.2021.696272

**Published:** 2021-07-01

**Authors:** Eva Martínez-Ispizua, Ángeles Calatayud, José Ignacio Marsal, Rubén Mateos-Fernández, María José Díez, Salvador Soler, José Vicente Valcárcel, Mary-Rus Martínez-Cuenca

**Affiliations:** ^1^Horticulture Department, Valencian Institute for Agricultural Research (IVIA), Valencia, Spain; ^2^Plants Genomics and Biotechnology Department, Institute for Plant Molecular and Cell Biology (IBMCP), Valencia, Spain; ^3^Biotechnology Department, Valencian Institute for the Conservation and Improvement of Agrobiodiversity (COMAV), Polytechnic University of Valencia, Valencia, Spain

**Keywords:** eggplant, landrace, nutraceutical value, antioxidants, phenotype, biodiversity

## Abstract

Given the little variability among commercialised eggplants mainly in developed countries, exploring, and structuring of traditional varieties germplasm collections have become a key element for extending ecotypes and promoting biodiversity preservation and consumption. Thirty-one eggplant landraces from Spain were characterised with 22 quantitative and 14 qualitative conventional morphological descriptors. Landraces were grouped based on their fruit skin colour (black-purple, striped, white, and reddish). Landraces B7, B20, and B24 were left out for their distinctive fruit characteristics. Wide variation for plant, leaf, flower, and fruit phenology traits was observed across the local landraces, and fruit descriptors were considered the most important ones. In a second experiment, landraces, B14, B16, and B17 were selected to determine fruit quality. By contemplating the benefits provided by antioxidants and sugars for human health, pulp antioxidant capacity, total phenolic, ascorbic acid, carotenoid, flavonoid, and total sugar content were determined. Significant differences were observed across these three landraces, and B14 was highlighted for its antioxidant properties, while B17 stood out for its high sugar content. B16 did not stand out for any traits. The results indicate the wide variability in eggplants for their phenotypic and nutritional characteristics, which emphasises the importance of traditional varieties as the main source of agricultural biodiversity.

## Introduction

Nutritional habits have vastly changed, and the consumption of fruit and vegetables has grown thanks to the abundance of health-promoting compounds found in them (Yahia et al., 2018). They are provided a wide range of minerals (Jiménez-Aguilar and Grusak, 2017), proteins (Raigón et al., 2008; Sedlar et al., 2021), fibre (Ciudad-Mulero et al., 2019) and antioxidants (Gürbüz et al., 2018; Karasawa and Mohan, 2018; Sidhu and Zafar, 2018). However, fruit and vegetables appreciation has mainly increased due to the beneficial effects associated with dietary antioxidants (Hussain et al., 2014).

Eggplant is a common annual vegetable crop grown in subtropical and tropical areas (Kaur et al., 2014). It is one of the most important vegetable crops, and 1.85 million cultivated hectares (ha) worldwide are used to grow it (with a production of 55 million tonnes). It has a huge economic impact in Africa, Europe, and especially Asia, which harvests more than 90% of the total eggplant production. It is particularly important in China and India. Spain is the world's tenth largest producer of this vegetable (Food and Agriculture Organization Faostat, 2019).

Although most commercial varieties are purple (Nothmann et al., 1976; Hanson et al., 2006), eggplants are known for being highly variable in fruit colour, shape, and size. A representative part of this diversity is found among traditional varieties. Landraces are crop varieties that have been differentiated by farmers through a historical selection process and they represent great genetic heritage as a source of agricultural biodiversity (Jain and Gupta, 2013). These local varieties are better adapted to specific agroclimatic conditions, and they are suitable for new agriculture kinds, such as organic production (Gonzalez-Cebrino et al., 2011; Ribes-Moya et al., 2018).

The fruit of the eggplant not only contains proteins, minerals, dietary fibre, minerals of interest as potassium, calcium, magnesium, sodium, iron (Quamruzzaman et al., 2020), but is also enriched in polyphenols, including phenolic acids such as chlorogenic acid, caffeic acid, and *p*-coumaric acid (Chumyam et al., 2013; Uscanga-Sosa et al., 2020), and flavonoids, including trace quantities of flavonols and a high content of various acylated and non-acylated anthocyanins specially in purple-coloured varieties (Koley et al., 2019). Also is appreciated for its content in other antioxidants as ascorbic acid (Fategbe et al., 2013) and vitamins, especially vitamin P (Dong et al., 2020), although has low provitamin A carotenoid content as compared to other Solanaceous crops such as tomatoes and peppers (Gürbüz et al., 2018). These bioactive compounds are responsible for higher functional properties of eggplant (Koley et al., 2019), as they neutralise reactive oxygen species (ROS) by reducing lipid peroxidation and damage to cellular organelles (Fategbe et al., 2013; Kaur et al., 2014), and provide antibacterial, anti-inflammatory, antiallergic, hepatoprotective, antithrombotic, antiviral, anticarcinogenic, and vasodilatory properties in humans (Rathee et al., 2009; Akanitapichat et al., 2010; Cushnie and Lamb, 2011; Grussu et al., 2011).

In relation to nutritional concerns, the eggplant (*Solanum melongena* L.) has become a highly appreciated crop. Of 120 vegetables evaluated to determinate their antioxidant capacity, eggplant was ranked in the top 10 for its oxygen radical absorbance capacity, mediated mainly by fruit's phenolic constituents (Stommel and Whitaker, 2003; Hanson et al., 2006). Nevertheless, a wide natural variation in antioxidant capacity has been found between eggplant landraces (Stommel and Whitaker, 2003; Hanson et al., 2006; Mennella et al., 2010). It is known that the quantity and quality of phenols present in fruit is conditioned by the environment, soil type, and also growing and storage conditions (Lee et al., 2004; Achouri et al., 2005; Luthria and Mukhopadhyay, 2006). Therefore, having a detailed description of the characteristics and nutraceutical quality of traditional eggplant varieties should attach considerable interest giving the high phenotype biodiversity that can be found in these landraces.

The main challenge of crop genetic selection lies in the reliability of available phenotypic data (Gosa et al., 2019). The rapid development of genomics, has offered crop breeders the ability to develop high yielding and stress tolerant plants, but the ability to acquire high yielding phenotypic data hinders this opportunity (Zhang and Zhang, 2018).

Currently, non-destructive phenotyping technologies, like hyperspectral imaging or multispectral fluorescence, are of great interest as they allow predicting the content of many nutraceuticals compounds without damaging the plant itself (Zarco-Tejada et al., 2009; Pu et al., 2015). Most of these facilities collect information in controlled environments using robotics and automatic image acquisition and analysis (Gosa et al., 2019). However, although this type of non-destructive technique can estimate a wide range of internal biochemical data in a short time, information on the ability of biochemical reflectance indices to quantify many compounds is still lacking (Sytar et al., 2017). Compared to hyperspectral studies, more progress has been made in fluorescence methods. The multi-channel fluorescence systems with multi-colour excitation have been significantly improved and the commercial devices became available and widely applied (Sytar et al., 2020). However, these technologies are still under active development (Yang et al., 2020).

This work seeks to revalue traditional eggplant varieties from the Valencian Community (Spain) as the biodiversity of the territory has been severely diminished by widely cultivated commercial hybrids. In this context, 31 eggplant landraces were selected from the plant resources stored in the genebank of the Institute for the Conservation and Improvement of Valencian Agrobiodiversity (COMAV, Valencia) and the Valencian Institute for Agrarian Research (IVIA, Valencia). Even if the majority of the selected eggplants had black-purple or striped skin, other less common varieties were included in the assay, since having a high degree of diversity was advantageous, both for their possible use in breeding programmes and for promoting their conservation. Once the phenotypic data had been collected, the nutritional and nutraceutical characteristics of three selected landraces were determined in order to gain benefits derived from their use.

## Materials and Methods

### Plant Material and Soil Experiment

The work herein presented is divided into two main experiments: one focuses on phenotypic characterisation and the other on nutritional quality. They were carried out in two consecutive years (2019 and 2020).

**Figure 1:**
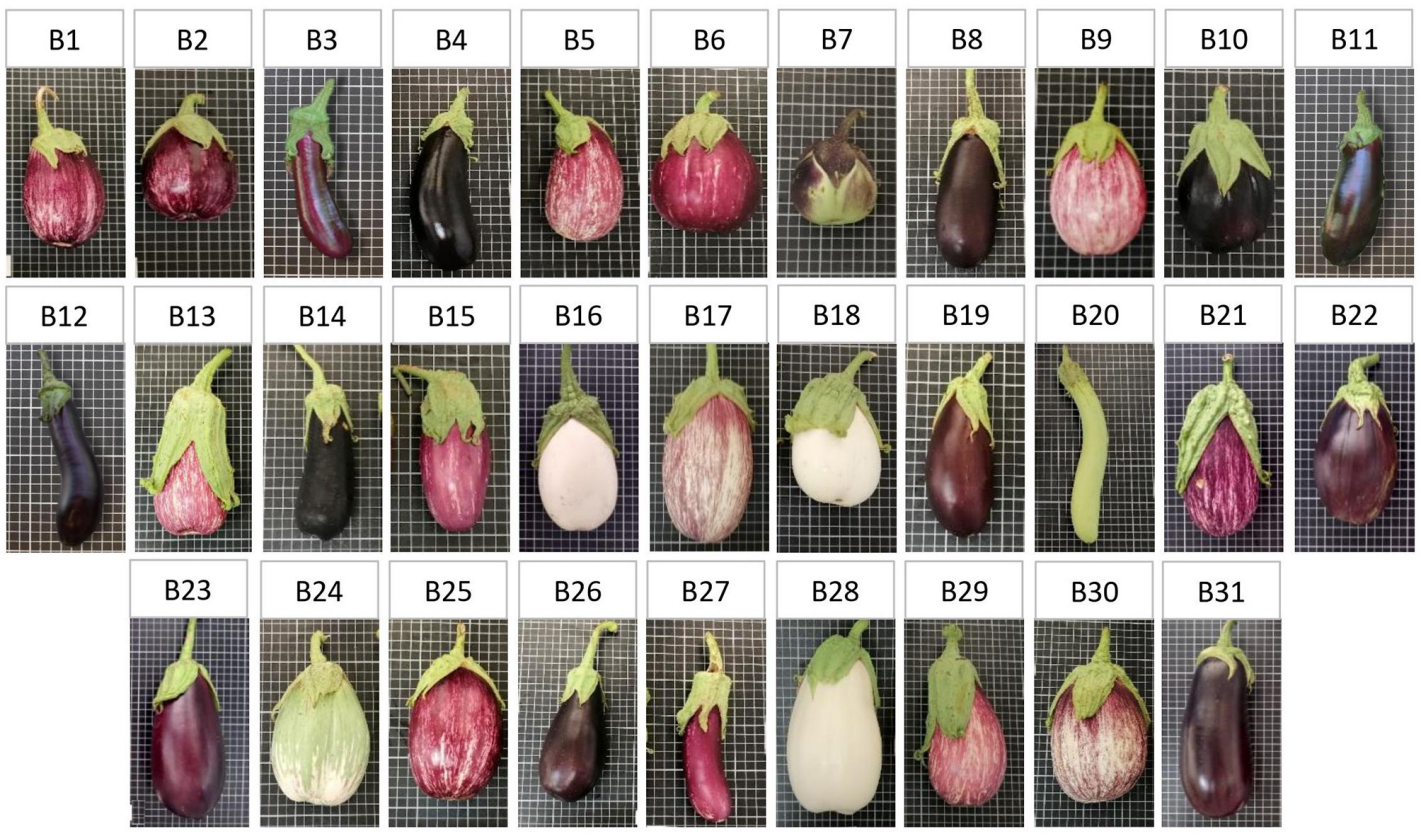
Pictures of the 31 cultivated eggplant landraces (*S. melongena* L.) provided by the Germplasm Banks from the COMAV and the IVIA (Spain). The size of the grid cells in the fruit pictures is 1 × 1 cm.

Seeds of eggplant landraces (*S. melongena L*.) were provided by the genebanks at the COMAV and the IVIA (Spain). The passport data are indicated in Table 1. Landraces were selected according to fruit colour and morphology to study as much phenotypic diversity as possible (Figure 1). In both years, the experiments were conducted from May to August in the experimental open-field of the IVIA located in Moncada (Valencia, Spain; 39° 35′ 22.3″ N, 0° 23′ 44.0″ W, 37 cm above sea level). Soil was sandy clay loan (clay: 21.2%; silt: 11.8%; sand: 67.0%), and organic matter was 0.61%, pH 7.8 at 25°C and EC_1:5_ at 25°C: 0.289 dS m^−1^.

**Table 1:** Abbreviation, germplasm collection code, group (based on eggplant skin colour, G1= black-purple, G2 = striped, G3 = white, G4 = reddish purple) and origin of the 31 eggplant landraces used in the study.

**Abreviation code**	**Genbank code**	**Group**	**Original location**
B1	BGV005769	G2	Alcira, Valenica, Spain^(a)^
B2	BGV005770	G2	Gandía, Valencia, Spain^(a)^
B3	BGV005771	G4	Gandía, Valencia, Spain^(a)^
B4	BGV005774	G1	Jaraco, Valencia, Spain^(a)^
B5	BGV005776	G2	Valencia, Spain^(a)^
B6	BGV005778	G2	Orihuela, Alicante, Spain^(a)^
B7	BGV005781	–	Benijofar, Alicante, Spain^(a)^
B8	BGV005780	'G1	San Fulgencio, Alicante, Spain^(a)^
B9	BGV005783	G2	Aspe, Alicante, Spain^(a)^
B10	BGV005784	G1	Novelda, Alicante, Spain^(a)^
B11	BGV005785	G1	Elche, Alicante, Spain^(a)^
B12	BGV005787	G1	Mutxamel, Alicante, Spain^(a)^
B13	BGV005788	G2	Benejama, Alicante, Spain^(a)^
B14	BGV005789	G1	Gandía, Valencia, Spain^(a)^
B15	BGV005790	G2	Orihuela, Alicante, Spain^(a)^
B16	BGV015751	G3	Alacuás, Valencia, Spain^(a)^
B17	BGV008284	G2	Moncada, Valencia, Spain^(a)^
B18	BGV015630	G3	Torreblanca, Castellón, Spain^(a)^
B19	BGV015745	G1	Benimasot, Alicante, Spain^(a)^
B20	BGV015762	–	Alcudia de Crespins, Valencia, Spain^(a)^
B21	BGV015847	G2	Onteniente, Valencia, Spain^(a)^
B22	BGV015763	G1	Onteniente, Valencia, Spain^(a)^
B23	BGV015848	G1	Onteniente, Valencia, Spain^(a)^
B24	BGV015849	–	Jaraco, Valencia, Spain^(a)^
B25	BGV015850	G2	Jaraco, Valencia, Spain^(a)^
B26	BGV015834	G1	Jaraco, Valencia, Spain^(a)^
B27	BGV015835	G4	Jaraco, Valencia, Spain^(a)^
B28	BGV015836	G3	Jaraco, Valencia, Spain^(a)^
B29	BGV014500	G2	Villarreal, Castellón, Spain^(a)^
B30	B-81	G2	Gandía, Valencia, Spain^(b)^
B31	B-76	G1	Alginet, Valencia, Spain^(b)^

*Plant material was provided by the:*

(a) *Valencian Institute for the Conservation and Improvement of Agrobiodiversity (COMAV, Spain);*

(b) *Valencian Institute for Agricultural Research (IVIA, Spain)*.

#### Experiment 1: Phenotyping Study

For the first-year experiment, 31 eggplant landraces were sown on 5 March 2019 and seedlings (8 plants per landrace) were planted on 2 May 2019. Plants were grown in single rows placed 120 cm apart leaving 60 cm between each plant. Irrigation of plants satisfied 100% of the crop evapotranspiration (ETc) as described in Penella et al. (2014) with a drip system. Nutrients were applied via the irrigation system at a rate (kg ha^−1^) of 200 N, 50 P_2_O_5_, 250 K_2_O, 110 CaO, and 35 MgO, as recommended by Maroto Borrego (2002). The average range of the minimum and maximum temperatures for the first-year experiment was 12–24°C for May, 15–28°C for June, 19–32°C for July, and 19–32°C for August (http://riegos.ivia.es/) (IVIA (Instituto Valenciano de Investigaciones Agrarias), 2021). Measurements were taken when fruits reached the commercial maturity, along July and August depending on the landrace (plants around 2.5–3 months after transplant). Data for plant, leaf, and flower traits were taken from eight independent plants, which gave 8 replicates per landrace. Fruit traits were measured in 10 different fruits which were representative of the landrace, which equals 10 replicates per variety.

#### Experiment 2: Fruit Quality Study

During the second-year experiment, landraces B14, B16, and B17 were chosen as being representative of the main fruit colour groups, namely, G1: black-purple, G2: striped and G3: white, to analyse fruit quality and to, thus, provide verified information on their added value and to facilitate their trade. Landraces from G4 group and outliers (B7, B20, and B24) were not considered due to their reduced number of representatives and unsuitable qualities for cultural practises and market observed in experiment 1 (high number of calix prickles, undesirable growth habit, low number of flowers per inflorescence, intense leaf pilosity…). Seeds were sown on 7 March 2020 and seedlings (10 plants per landrace) were planted on 13 May 2020. Agronomic culture practises were similar as in the first-year experiment. The average range of the minimum and maximum temperatures was 11–31°C for May, 14–31°C for June, 18–33°C for July and 19–34°C for August (http://riegos.ivia.es/) (IVIA (Instituto Valenciano de Investigaciones Agrarias), 2021). Fruits were harvested when reached the commercial maturity, along July and August depending on the landrace (plants around 2.5–3 months after transplant). For each case, two fruit samples were taken from ten independent plants, which gave 20 replicates per landrace.

### Agromorphological Characterisation and Data Collection

The quantitative and qualitative agromorphologic data from thirty-six phenotypic traits (Table 2) measured in plants, stem, leaves, flowers, and fruit were scored and classified according to the International Board for Plant Genetic Resources descriptors (IBPGR 1990) for eggplant.

**Table 2:** List of the descriptors used for phenotyping according to the International Board for Plant Genetic Resources descriptors (IBPGR 1990) for eggplant.

**Descriptors**	**Unit/Scale**
**Quantitative**	
**Plant morphology**	
Length	cm
Width	cm
Branch density	1.Very scarce (≤ 2) 3.Scarce (~5) 5.Intermediate (~10) 7.Dense (~20) 9.Very dense (>30)
**Leaf morphology**
Length	cm
Width	cm
Pedicel length	mm
Pedicel thickness	mm
Colour	L*
	a*
	b*
Dented leaf blade	1.Very weak 3.Weak 5.Medium 7.Strong 9.Very strong
Blistering	1.Very weak 3.Weak 5.Medium 7.Strong 9.Very strong
Pilosity (per cm^−2^)	1. (<20) 3. (20–50) 5. (50–100) 7. (100–200) 9. (> 200)
**Flowers**	
Number per inflorescence	
**Fruit**	
Number of colours in commercial maturity	
Length	cm
Width	cm
Length/width	
Weight	g
Calyx length	cm
Calyx lenght total lenght ratio	%
Number of calyx prickles	
**Descriptors**	**Unit/Scale**
**Qualitative**	
**Plant morphology**	
Plant growth habit	3.Erect 5.Intermediate 7.Postrate
Relative internode length	1.Short 2.Medium 3. Long
Anthocyanin intensity pigmentation	0.Absent 1.Weak 2.Medium 3.Strong
Pilosity	1.Weak 2.Medium 3.Strong
**Leaf morphology**	
Prickels (upper surface)	0. Absent 1. (1–2) 3. (3–5) 5. (6–10) 7. (11–20) 9. > 20
**Flower morphology**	
Corolla colour	1.Greenish white 3.Light White 5.Pale violet 7.Bright violet 9.Bluish violet
**Fruit morphology**	
Longitudinal shape	1.Globular 2.Ovoidal 3.Ovobal 4. Pear shape 5.Deck shape 6.Ellipsoid 7.Cylindrical
Curvature	1.Straight 3.Slightly curved 5.Curved 7.Snake shape 8.Sickle shape 9.U shape
Cross section shape	1.Circular, without grooves 3.Elliptical, without grooves 5.Few grooves (~4) 7.Lots of grooves (~8) 9.Very irregular
Apex shape	3.Protuberant 5.Rounded 7.Depressed
Colour distribution at commercial maturity	1.Uniform 3.Mottled 5.Compensated 7.Striped
Pulp colour	1.White 3.Intermediate 5.Green
Predominant colour in commercial maturity	1.Green 2.Milk white 3.Dark yellow 4.Fire red 5.Redish purple 6.Greyish lilac 7.Purple 8.Black purple 9.Black
Secondary colour (if any)	1.Green 2.Milk white 3.Dark yellow 4.Fire red 5.Redish purple 6.Greyish lilac 7.Purple 8.Black purple 9.Black

#### Leaf and Fruit Peel Colour

The colour of eggplant leaves was determined by placing the laboratory standard colorimeter (Minolta Colorimeter model CR-400, Osaka, Japan) on the central part of the adaxial face. Two independent colour measures were taken in each plant, which gave 16 data per landrace. L^*^ (lightness), a^*^ (red/green chromatic coordinates) and b^*^ (yellow/blue chromatic coordinates) measures were recorded in order to determine leaf colour. L^*^ a^*^ b^*^ standard for colour measurement was chosen as it is perceptually uniform and device-independent (Mendoza et al., 2006). Eggplant peel colour was assigned by researchers visually, being thus considered a qualitative trait.

### Fruit Quality Determinations

The percentage of dry weight (DW), pulp colour, antioxidant capacity, and total phenolic, flavonoid, ascorbic acid, carotenoid and sugar contents, were analysed in the mid-part of the pulp of harvested fruits in B14, B16, and B17 to determine if there were significant differences among them.

#### Fruit Dry Material

In order to establish the percentage DW in fruits, the fresh weight (FW) of eggplants was recorded. Samples were dried at 65°C for 72 h in a laboratory oven. The DW percentage was calculated as (DW/FW) × 100.

#### Pulp Colour

Fruit slides (10 mm wide Zaro et al., 2014b) were cut transversally in the central part of the eggplant and colour in the inner pulp was measured by laboratory standard colorimeter (Minolta Colorimeter model CR-400, Osaka, Japan). One measure in the central part of the sample was taken in each fruit, which gave 20 data per landrace. L^*^ (lightness), a^*^ (red/green chromatic coordinates), and b^*^ (yellow/blue chromatic coordinates) measures were recorded in order to determine pulp colour immediately after eggplants were cut (Concellón et al., 2012). L^*^ a^*^ b^*^ standard for colour measurement was chosen as it is perceptually uniform and device-independent (Mendoza et al., 2006).

#### Nutraceutical Compounds and Antioxidant Capacity

##### Sample Extract

Nutraceutical compounds and antioxidant capacity were analysed in the pulp of eggplant fruit. Samples were peeled, cut into pieces and homogenised (Polytron PT 3100, Kinematica AG.,) at 15,000 g for approximately 1 min. Final extracts were divided into aliquots of 2 g, frozen in liquid nitrogen and stored at −80°C until further determinations were made.

##### Antioxidant Capacity Measurements

Antioxidant capacity was measured following the method reported by Brand-Williams et al. (1995) with a few modifications. The sample extract (1 g) was homogenised in 4.0 mL 80% (v/v) methanol, incubated in an ultrasonic bath (Ultrasonic cleaner, Fungilab) at medium intensity for 30 min and then vortexed. Samples were centrifuged at 10,000 g at 4°C for 15 min. Then 10 μL of the extract were mixed with 990 μL of a solution composed of 3.12 × 10^−5^ M of 2,2-diphenyl-1-picrylhydrazyl (DPPH) in 80% methanol. The decrease in absorbance at 515 nm contrasted with a blank solution containing 80% methanol with no extract after a 30-min reaction time at room temperature and in the dark using a spectrophotometer (Uvikon XS, Bio-Tek). Antioxidant capacity was expressed as the 149 percentage reduction of the initial DPPH absorption in extracts.

##### Total Phenolic Content

Phenolic content was analysed as described by Dewanto et al. (2002) with some adjustments. The sample extract (1 g) was mixed with 4.0 mL of 80% (v/v) methanol, vortexed and incubated in an ultrasonic bath (Ultrasonic cleaner, Fungilab) at medium intensity for 30 min. Samples were centrifuged at 10,000 g at 4°C for 15 min. The total phenolic concentration was determined following the procedure of Singleton and Rossi (1965) based on the Folin-Ciocalteu colorimetric method. Then 10 μL of the supernatant were mixed with 115 μL of distilled water, 125 μL of Folin-Ciocalteu reagent (Sigma-Aldrich, Co.) and 1.25 mL of NaHCO3 (7%). Afterwards the mix was incubated at room temperature for 90 min in complete darkness. The absorption of the solution was measured at 760 nm in a spectrophotometer (Uvikon XS, Bio-Tek). A blank solution with no extract was used for calibration. Total phenolic concentration was compared to a standard curve using gallic acid (120–600 mg L^−1^). Total phenolic content was expressed as mg gallic acid equivalent (GA) g^−1^ FW.

##### Total Flavonoid Content

Flavonoid content was measured following the method reported by Du et al. (2009) with some modifications. Briefly, 1 g of sample extract was homogenised in 4.0 mL of 80% (v/v) methanol, incubated in an ultrasonic bath (Ultrasonic cleaner, Fungilab) at medium intensity for 30 min and then vortexed. Samples were centrifuged at 10,000 g at 4°C for 15 min. Then 0.3 mL of the supernatant were mixed with 3.4 ml of 30% ethanol, 0.15 ml of NaNO_2_ 0.5 M, and 0.15 mL of AlCl_3_. Next 6H_2_O 0.3 M was added and vortexed. Samples were incubated for 5 min at room temperature. Afterwards 1 mL of NaOH 0.1 M was added to the mixture. The absorption of solution was measured at 506 nm in a spectrophotometer (Uvikon XS, Bio-Tek). Total flavonoid concentration was compared to a standard curve using rutin (Merck Co.) as the standard (4.7–300 mg L^−1^). Flavonoid content was expressed as mg rutin equivalent g^−1^ FW.

##### Ascorbic Acid Concentration

Ascorbic acid content was spectrophotometrically determined according to Kampfenkel et al. (1995). The sample extract (0.3 g) was mixed with 2 mL of 6% (w/v) TCA (trichloroacetic acid). Samples were centrifuged at 10,000 g for 3 min. Next 0.05 mL of the supernatant were mixed with 0.05 mL of 10 mM DTT and 0.1 mL of 0.2 M phosphate buffer (pH 7.4). Samples were incubated for 15 min at 42°C. Subsequently, 0.05 mL of 0.5% (w/v) NEM (N-ethylamide) were added to the mix and incubated for 1 min at room temperature. Later 0.25 mL of 10% (w/v) TCA, 0.2 mL of H_3_PO_4_, 0.2 mL of 4% (w/v) 2,2'-dipyridyl, and 0.1 mL of 3% (w/v) FeCl_3_ were added to the previous solution. The mixture was incubated in a water bath for 40 min at 42°C. The absorption of solution was measured at 525 nm in a spectrophotometer (Uvikon XS, Bio-Tek). Ascorbic acid was expressed as mg AsA 100 g^−1^ FW.

##### Carotenoid Concentration

The carotenoid concentration was determined spectrophotometrically as reported by Porra et al. (1989). The sample extract (0.3 g) was mixed with 1.5 mL of 80% acetone (v/v) and centrifuged at 7,000 g for 10 min. The supernatant was used for the analysis. The absorption of solution was measured at 663, 648, and 470 nm in a spectrophotometer (Uvikon XS, Bio-Tek) and 80% acetone (v/v) was used for the blank solution. The carotenoid concentration of samples was calculated using Equation (1), and then expressed as μg g^−1^ FW:

(1) $$\begin{array}{l} \text{Carotenoid}s({\text{μ g mL}}^{–\text{1}})=[(1000\times {\text{Abs}}_{470}-1.82\times \text{Chl a})\\ -(85.02\times \text{Chl}\;\text{b})]/198 \end{array}$$

Where Chl a and Chl b were the chlorophyll a and b contents, respectively, and were calculated by Equations (2) and (3); Abs is the absorption of samples at a specific wavelength (nm):

(2) $$\text{Chl a}({\text{μ g mL}}^{\;–\text{1}})=12.25\times {\text{Abs}}_{663}-2.55\times \;{\text{Abs}}_{648}$$

(3) $$\text{Chl b}({\text{μ g mL}}^{\;–\text{1}})=20.31\times {\text{Abs}}_{648}-4.91\times \;{\text{Abs}}_{663}$$

#### Total Soluble Sugar Content

Soluble sugar content was spectrophotometrically determined according to Calatayud et al. (2008) with several modifications. The sample extract (0.3 g) was mixed with 15 mL of 80% ethanol (v/v), which was previously heated. The mixture was incubated in a water bath for 10 min at 85°C and then vortexed. Samples were centrifuged at 10,000 g at 23°C for 10 min. The resulting supernatant was reserved in a flask. This same process was repeated 2 more times by adding hot ethanol to the mixing tube. The ethanol present in the reserved supernatant was then evaporated by a rotary evaporator (R-210, Buchi) at 50°C. The sugar concentrate was diluted in 100 mL of distilled water and filtered to be reserved in a volumetric flask for 24 h at 4°C. Next 0.5 mL of this solution was mixed with 2 mL of distilled water and placed on ice. Once cooled, 5 mL of 4 μM anthrone (Acros Organics B.V.B.A.) solution, diluted in 96% (v/v) sulphuric acid, were added to each tube. Samples were incubated in a water bath for 7.5 min at 85°C and then placed on ice for 30 min. The absorption of solution was measured at 630 nm in a spectrophotometer (Uvikon XS, Bio-Tek). Total sugar concentration was compared to a standard curve using a diluted (1:25) stock solution of 55.6 μM glucose and 70 μM sodium benzoate as the standard. Total sugar content was expressed as g glucose equivalent 100 g^−1^ FW.

### Statistical Analysis

The results obtained from the evaluated parameters underwent a one-way ANOVA analysis in Statgraphics Centurion XVII (Statistical Graphics Corporation 2014) using the selected landraces as the factor of analyses. The results were expressed as the mean ± standard deviation (SD). The means were accepted as being significantly different at a 95% confidence interval (*p* ≤ 0.05).

Principal component analysis (PCA) was run for the standardised values using pairwise Euclidean distances among accession means to determinate any relations between genotypes. The extracted eigenvalues, and the relative and cumulative proportions of the total variance explained by the first three components, were calculated. Two-dimensional (2D) scatter plots (the first vs. the second and the first vs. the third principal components) were prepared based on a distance matrix for the principal components to visualise the relation that explained traits. For the PCA analysis, the phenotypic data pertaining to the 31 landraces was considered together.

Furthermore, two correlation analyses, in which the individual samples of each accession were subjected to linear regression and the correlation coefficients (r), were completed among the: (1) selected phenotypic quantitative traits of each landrace (*n* = 31); (2) dry weight, pulp colour and antioxidant traits of the selected landraces (B14, B16, B17).

## Results

### PCA Analysis of Phenotyping Traits

The PCA analysis and those eigenvalues above 1 reflected a different pattern in the correlation of eggplants (Table 3). Nine principal components were determined, which described around 80% of the variability between landraces. Here it is only shown the distribution of landraces based on the most significant principal components; the first, second and third components of the PCA, which, respectively, accounted for 23.33, 16.73, and 12.04% of the total variation for the studied traits.

**Table 3:** Correlation coefficients for each morphological trait for the three first principal components, eigenvalue, and relative and cumulative proportion of the total variance explained by these components in the collection of the 31 eggplant landraces.

	**First principal component**	**Second principal component**	**Third principal component**
Pl Growth habit	0.216	0.194	
Pl Branch density		−0.159	−0.199
Pl Height	−0.225		
Pl Width			−0.164
S Relative internode length		−0.173	−0.230
S Anthocyanin pigments intensity		−0.334	
S Pilosity		−0.227	
L Puffiness	0.211		
L Dental leaf blade		0.291	
L Thorn presence			−0.339
L Pilosity			
L Length			−0.240
L Width	0.213		−0.294
L Pedicel length		−0.180	−0.274
L Pedicel thickness			−0.351
L Colour parameter L*		0.304	
L Colour parameter a*		−0.309	
L Colour parameter b*		0.342	
Fl Corolla colour	−0.204		
Fl Number per inflorenscence			
Fr Longitudinal shape	−0.280		−0.176
Fr Curvature	−0.214		−0.209
Fr Cross section shape			0.208
Fr Apex shape	0.260		
Fr Colour distrib. at maturity	0.253		
Fr Pulp colour	−0.257		
Fr Colour predominant maturity		−0.272	
Fr Secondary colour (if any)	0.174		
Fr Number of colours	0.193		
Fr Length	−0.188	0.204	−0.222
Fr Width	0.280		
Fr Length/width ratio	−0.252		−0.215
Fr Weight	0.212		
Fr Calyx length			
Fr Calyx lenght/total lenght	0.172		0.186
Fr Calyx prickles number	0.182		
*Eigenvalue*	8.400	6.022	4.336
*Variance explained (%)*	23.334	16.728	12.043
*Cumulative variance explained (%)*	23.334	40.062	52.105

*Only the correlations with absolute 0.150 ≤ are listed. Pl, Plant; S, Stem; L, Leaf; Fl, Flower; Fr, Fruit*.

The first component principally correlated with fruit traits. All the correlations were moderate, and the strongest positive relations were observed with fruit width, apex shape, and skin colour distribution upon maturity. Negative correlations were found with fruit longitudinal shape, pulp colour, and the length-width ratio. Therefore, the darkest and longest eggplants with obscure pulp were placed to the left of the plot, while the widest and lightest eggplants with a striped/mottled skin colour distribution and a whitish pulp were placed to the right of the plot. In line with this, when analysing the second component, the highest correlations were recorded for the leaf and stem traits. In particular, positive correlations were established with colour parameters L^*^ and b^*^ and dental leaf blade, while negative correlations were found for stem anthocyanin pigments intensity and leaf colour parameter a^*^, among others. So the landraces whose leaves had an intense light-green colour and with very lobed margin were placed in the upper part of the plot, while the landraces with dark-green leaves with soft margins and absence of anthocyanins on the stem were placed in a lower position. The third component of the PCA analysis showed that the correlations with fruit descriptors followed the same pattern as that observed for the first component. In contrast, negative correlations were established with some leaf descriptors: width, presence of prickles, pedicel thickness, and pedicel length. In each group described according to the fruit criteria, those landraces with wider leaves, lacking prickles, and long pedicels were placed in the upper position in the plot.

**Figure 2:**
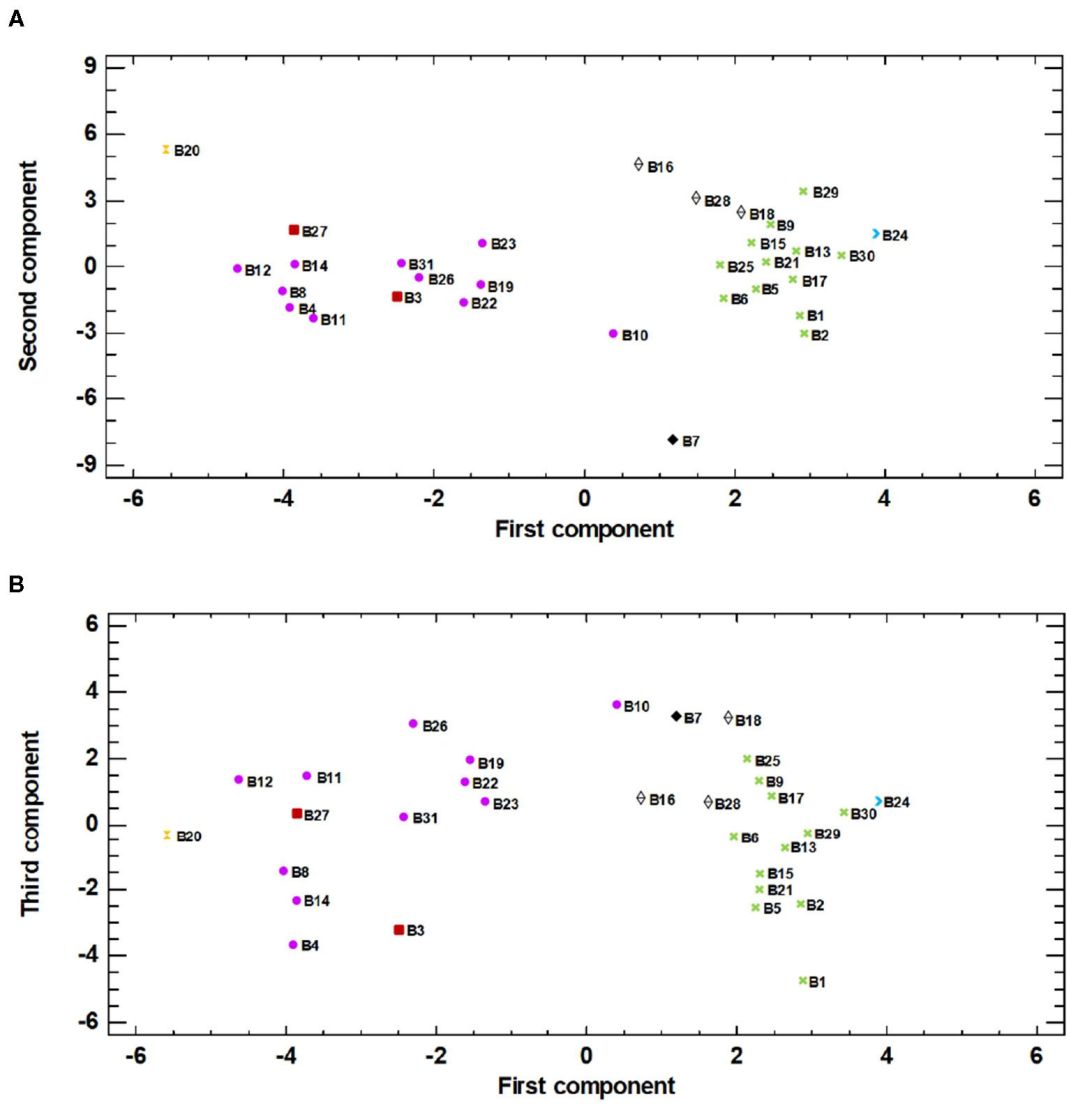
The principal component analysis (PCA) for the 31 eggplant accessions based on the traits used for phenotyping represented in **(A)** the two first components (first component, X-axis; second component, Y-axis) of the PCA (23.34 and 16.73% of total variation, respectively) and **(B)** the first and third components (first component, X-axis; third component, Y-axis) of the PCA (23.34 and 12.04% of total variation, respectively). Groups, arranged based mainly on fruit skin colour (G1 black-purple, G2 = striped, G3 = white, G4 = reddish purple), are represented in the plot: G1 (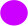), G2 (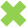), G3 (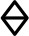), and G4 (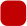). Outliers B7 (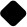), B20 (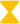), and B24 (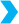) are also expressed in the figure.

The projection on the PCA plot (Figure 2) showed how accessions spread widely over the area. In general, the landraces that were similar in fruit skin colour and shape were placed together, which highlights the importance of both traits. According to this information, several groups were arranged based mainly on fruit skin colour: G1 = black-purple, G2 = striped, G3 = white, G4 = reddish purple. The dark and striped skin eggplants were clearly separated in the plot. The white landraces remained close to the striped ones because of their globular shape, while the reddish ones remained near the black-purple ones given their dark skin colour and elongated shape. Notwithstanding, it was considered necessary to differentiate groups G3 and G4 for their distinctive fruit traits. Landraces B7, B20, and B24 were not included in any of these groups in PCA analysis because of their distinctive fruit morphology.

In the plot corresponding to first and second components (Figure 2A), the landraces from G2, G3, and B24 were located furthest to the right according to the correlations determined in the first component. In contrast, landrace B20 was located on the left of the graph because its fruits ranked first for the both fruit-length and width-length ratios. B20 was located in the top position of the plot because its leaves obtained higher values for colour parameters L^*^ and b^*^, and the lowest value for colour parameter a^*^. In contrast, landrace B7 remained at the bottom of the plot given the strong anthocyanin pigmentation on the stem in addition to having the lowest values for leaf parameters L^*^ and b^*^. G1 and G4 were also located on the left of the plot, principally for their elongated shape and their uniform-mottled skin colour distribution. B10 was slightly separated for having the lowest fruit length/width ratio.

The plot projecting the landrace distribution based on the first and third principal components (Figure 2B) did not differ that much from the previous one. In this case however, the varieties of each group appeared somewhat more dispersed given the leaf morphology effect. Landraces B10, B7, B18, and B26 appeared at the top of the graph. B10 stood out for its globous leaves, and B7 and B18 lacked prickles on leaves and displayed a very short pedicel length. B26 stood out for presenting very thin leaves with fine pedicels. Conversely, B1 was located at the bottom of the plot for its wide leaves with thick pedicels and for also presenting the most marked presence of prickles.

### Phenotypic Differences Between Eggplant Landraces

**Figure 3:**
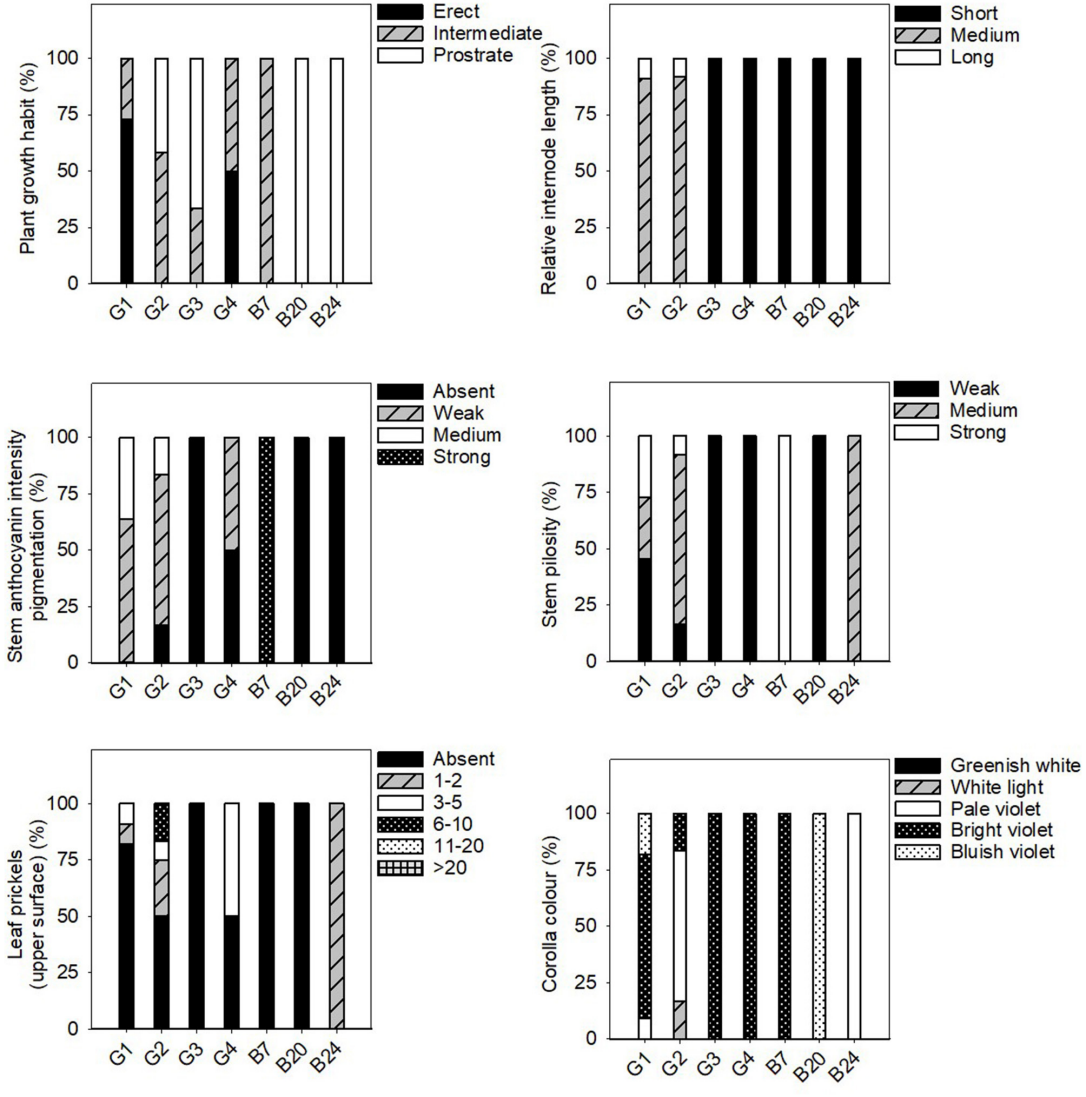
Frequency distribution (%) of the stem, leaf and flower qualitative traits in the 31 eggplant landraces in each group (G1, G2, G3, G4) and B7, B20, and B24. Measurements were taken when fruits reached the commercial maturity. Data for plant, leaf and flower traits were measured from eight independent plants, which gave 8 replicates per landrace.

**Figure 4:**
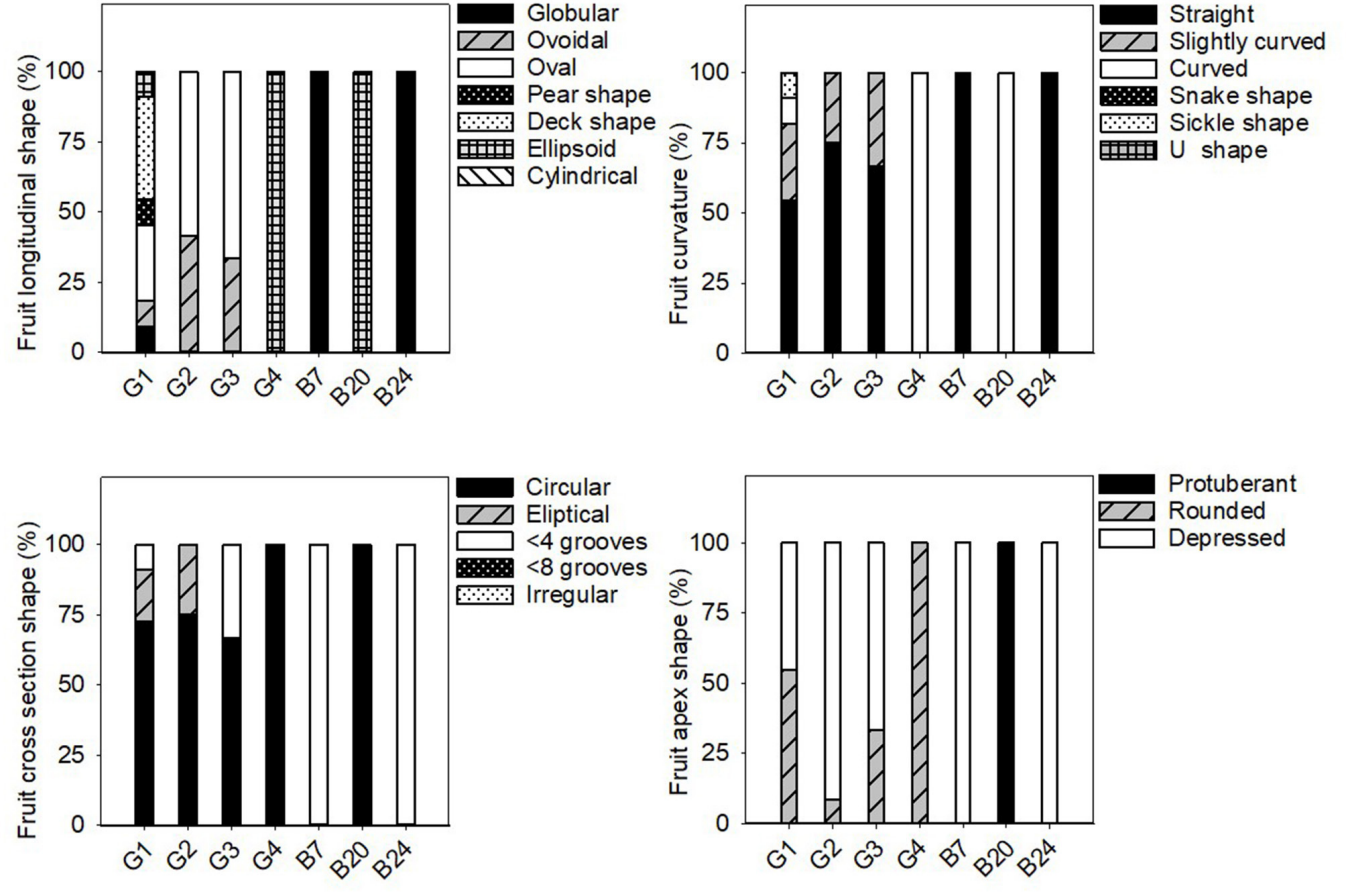
Frequency distribution (%) of the fruit qualitative traits related to fruit shape and size in the 31 eggplant landraces in each group (G1, G2, G3, G4) and B7, B20, and B24. Measurements were taken when fruits reached the commercial maturity. Data for fruit traits were measured from 10 different fruits which were representative of the landrace.

**Figure 5:**
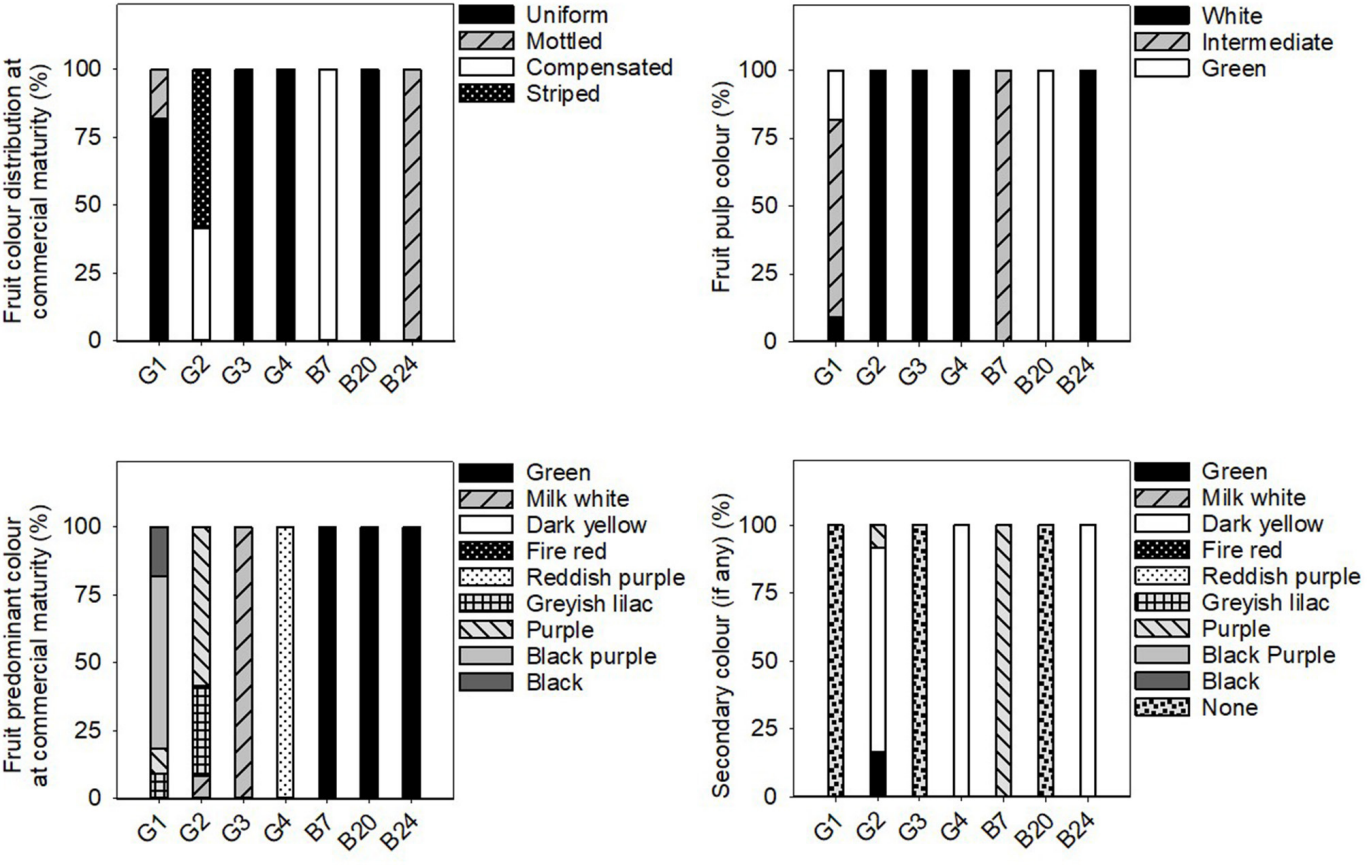
Frequency distribution (%) of the fruit qualitative traits related to fruit colour in the 31 eggplant landraces in each group (G1, G2, G3, G4) and B7, B20, and B24. Measurements were taken when fruits reached the commercial maturity. Data for fruit traits were measured from 10 different fruits which were representative of the landrace.

Significant differences were found among the average values of all the eggplant groups for the majority of the considered quantitative traits (Table 4). The individual data for each landrace of these groups is shown in the Supplementary Tables 1, 2. All the qualitative data is found in Figures 3–5.

**Table 4:** Variation parameters for the conventional morphologic quantitative descriptors in the 31 local eggplant landraces cultivated in Spain. Statistics were performed by the formed groups based on fruit skin colour; G1 = black–purple, G2 = striped, G3 = white, G4 = reddish purple.

**Landrace**	**G1**				**G2**			
**Descriptors**	**Mean**	**Range**	**CV (%)**	**F–ratio**	**Mean**	**Range**	**CV (%)**	**F-ratio**
**Plant**								
Length	94.92	58–128	16.85	15.3***	78.18	58–109	13.48	9.10***
Width	108.94	80–155	13.64	2.97**	113.75	89–143	11.02	3.88***
Branch density	5.54	4.90–7	9.93	11.06***	5.60	5–8	10.81	3.84***
**Leaf**								
Length	20.22	11–27.90	14.10	2.58*	21.58	14.5–30	14.33	1.84
Width	12.71	8.50–21	21.26	5.17***	16.81	10–24.50	18.50	4.63***
Pedicel length	8.72	3.50–16	31.70	4.97***	8.82	4–15	23.14	4.41***
Pedicel thickness	5.53	3.35–9.10	19.04	5.32***	7.00	4–10.75	21.53	26.75***
Dented leaf blade	3.44	1–7	48.90	28.95***	6.23	4–8	15.89	18.33***
Blistering	2.90	1–5	24.85	16.05***	3.58	1–7	55.61	170.99***
Pilosity (per cm^−2^)	5.80	3–7	23.91	79.8***	5.42	3–7	26.80	200<^***^
Leaf L*	29.96	26.22–34.58	4.99	5.94***	29.68	23.96–33.71	6.09	7.76***
Leaf a*	−6.55	−9.87 to −4.83	|12|	9.01***	−7.09	−8.68 to −4.77	|11.78|	14.2***
Leaf b*	9.54	7.26–12.93	11.88	9.5***	9.96	6.80–13.28	11.83	5.9***
**Flowers**								
Number per inflorescence	1.27	1–2	35.15	24.74***	1.36	1–3	38.92	56.26***
**Fruit**								
Number of colours in commercial maturity	1.31	1–2	35.48	134.43***	2.03	1–3	10.21	1.9*
Length	14.95	7.30–26	27.16	19.92***	13.42	8.50–19.50	18.63	16.04***
Width	6.50	3.98–11.15	25.44	34.97***	8.17	5.48–11.69	18.46	9.55***
Length/width ratio	2.52	1.02–4.82	31.64	11.98***	1.67	1.08–2.92	22.31	13.03***
Weight	216.07	111.27–581.65	47.40	18.14***	328.34	112.04–808.30	43.63	8.87***
Calyx length	7.60	4.40–15.20	24.90	8.58***	8.84	4.30–20.70	40.88	10.59***
Calyx lenght total lenght ratio	53.58	24–102.68	38.30	35.33***	64.70	28.21–152.21	32.76	4.80***
Number of calyx prickles	8.25	0–38	107.17	3.37***	20.94	0–73	80.06	7.86***
**Landrace**	**G3**				**G4**			
**Descriptors**	**Mean**	**Range**	**CV (%)**	**F-ratio**	**Mean**	**Range**	**CV (%)**	**F-ratio**
**Plant**								
Length	72.71	51–98	21.20	28.04***	93.78	86–109	6.71	2.14
Width	99.38	57–126	15.89	11.53***	99.81	84–118	9.85	3.61
Branch density	5.04	3–7	15.22	6.45**	6.00	5–7	12.17	14.00**
**Leaf**								
Length	20.37	18–24.50	10.39	4.58	20.25	16–24.60	12.78	8.90**
Width	15.14	10.90–22	18.06	5.10	14.31	10–20.50	22.78	18.26***
Pedicel length	6.95	5.50–9	15.74	2.93	7.38	5–10	20.04	13.37**
Pedicel thickness	6.52	5–7.90	12.48	3.64*	6.00	4.40–8.60	23.27	32.62***
Dented leaf blade	4.67	4–6	10.35	3.71*	4.63	2–7	41.68	43.81***
Blistering	5.32	4–8	26.20	12.52***	3.31	3–5	18.18	5.65*
Pilosity (per cm^−2^)	5.35	3–7	33.17	82.29***	5.63	3–7	28.20	56.47***
Leaf L*	30.40	26.05–34.58	6.29	5.29**	29.68	27.27–33.25	4.78	5.72*
Leaf a*	−7.57	−10.10 to−5.86	|11.48|	9.46***	−6.74	−9.17 to −5.23	|12.62|	0.16
Leaf b*	10.84	8.61–14.29	12.77	5.04*	9.34	7.74–12.36	12.22	2.31
**Flowers**								
Number per inflorescence	1.65	1–2.10	29.45	7.82*	1.23	1–2	35.63	2.54
**Fruit**								
Number of colours in commercial maturity	1.00	–	0.00	1.00	1.00	–	0.00	1.00
Length	13.79	10–21	16.23	3.57*	15.83	13.4–22	14.18	1.71
Width	8.54	5.86–12.18	20.74	34.78***	4.10	3–5.2	17.89	0.35
Length/width ratio	1.68	1.12–2.36	22.17	29.24***	4.03	2.91–4.92	13.77	0.06
Weight	349.17	121.90–791.40	37.53	9.57***	126.15	66.3–208.53	30.67	0.69
Calyx length	8.62	5.50–12.50	23.06	10.89***	8.23	4.7–12.5	22.87	0.29
Calyx lenght total lenght ratio	63.37	35.26–100	26.15	5.82***	49.17	40.46–55.97	9.97	20.01**
Number of calyx prickles	22.73	0–57	81.49	7.44**	5.89	0–16	86.37	0.20
**Landrace**	**B7**			**B20**			**B24**	
**Descriptors**	**Mean**		**Range**	**Mean**		**Range**	**Mean**	**Range**
**Plant**								
Length	83.13		79–91	86.00		66–106	88.83	81–105
Width	99.13		93–103	105.17		98–108	135.57	121–150
Branch density	5.63		5–6	5.00		–	6.00	–
**Leaf**								
Length	22.50		19.50–26	19.70		18–21.90	20.00	17–25
Width	15.44		12–17.50	13.86		12.50–15	14.79	13.50–17
Pedicel length	7.75		6–11	5.64		5–6	9.00	4.90–14
Pedicel thickness	5.80		5.15–6.70	5.76		4.90–6.08	5.41	4.70–6.30
Dented leaf blade	5.00		–	5.14		4–6	6.00	–
Blistering	1.00		–	5.43		4–6	3.29	3–4
Pilosity (per cm^−2^)	7.00		–	5.00		–	7.00	–
Leaf L*	25.16		19.67–30	32.49		28.26–36.95	31.30	28.84–33.45
Leaf a*	−3.72		−8.19 to 0.61	−7.99		−9.22 to −6.26	−7.61	9.55 to −6.46
Leaf b*	7.18		3.36–11.44	12.39		8.23–15.10	10.73	9.89–11.13
**Flowers**								
Number per inflorescence	2.63		2–3	1.00		–	1.00	–
**Fruit**								
Number of colours in commercial maturity	1.00		–	2.00		–	3.00	–
Length	6.14		5.60–6.80	22.11		14.70–26.90	11.71	8.10–14.30
Width	7.11		6.35–8.16	3.72		3.05–4.44	9.04	7.36–9.81
Length/width ratio	0.87		0.75–0.95	6.17		5.77–7.15	1.36	1.09–1.64
Weight	112.89		83.37–146.80	120.22		70.27–186.02	341.42	231.83–465.69
Calyx length	6.94		6.20–7.70	5.89		5.40–6.50	8.00	5.80–11.30
Calyx lenght total lenght ratio	110.86		95.59–120.69	25.98		23.66–28.93	68.89	50.74–93
Number of calyx prickles	2.63		0–5	3.50		0–8	4.50	0–11

Data belonging to outliers (B7, B20, B24) is also shown. Values represent the mean, range, coefficient of variation (CV, %), F–ratio and significance (***, **, * indicate significance at p < 0.001, p < 0.01, p < 0.05), for the conventional morphological descriptors studied in cultivated eggplants (n = 8 for plant, leaf, and flower traits and n = 10 for fruit traits).

In general, the fruit purple-black eggplant varieties (G1) were characterised by an erect plant growth habit, medium relative internode length, weak stem anthocyanin pigmentation, strong leaf pilosity and bright violet flowers. The fruits themselves stand out for their oval, pear or deck shape, and were much longer than they were wide, with a rounded or depressed apex, no curvature, white to greenish pulp and their long, but not very prickly calyx.

The landraces grouped for their striped fruit-skin (G2) were mostly characterised by an intermediate prostrate growth habit, medium relative internode length, large hairy leaves and pale flowers. Their fruits were elongated, non-curved with a depressed apex, white pulp, many prickles on the calyx and they were considerably heavy. Dark yellow and purple colours predominated on their skin, along with a striped or compensated colour distribution.

Three of the landraces in this study had white skin fruits (G3) and generally presented short internodes, prostrate growth habit, weak stem pilosity, absence of anthocyanins on the stem and many bright violet flowers. The aforementioned fruits had white pulp, many prickles, an oval-ovoidal shape and no curvature.

Only two accessions that produced reddish-purple fruit were detected (G4). These plants presented erect-intermediate growth habit, dense branching, short internodes, weak stem pilosity, and bright violet flowers. Their fruits were elongated, ellipsoid-shaped and curved, and they presented a rounded apex, white pulp and a few prickles on the calyx.

Landraces B7, B20, and B24 were not included in any of the groups because of their distinctive fruit typology. Variety B7 had globular fruits that were equally green and purple in colour, and were very light and small in size. This landrace was also notable for its high anthocyanin content on its hairy stem. The B20 entry had very elongated ellipsoid fruits that were green in colour with no prickles on the calyx. Finally, landrace B24 had mostly green and globular-shaped fruits, but with yellow stripes on the lower part. They also characterised for their white pulp and elevated weight.

### Correlation Among the Selected Agro-Morphological Quantitative Traits

Correlation analyses were carried out to estimate the relation between the most important quantitative traits (Table 5). The pairwise coefficients showed a positive correlation and a statistical significance for 15 pairs of traits of the 55 studied ones. The most representative positive relations were observed between fruit width vs. weight, fruit ratio vs. fruit length and leaf length vs. width. Statistically significant negative correlations for pairs of traits were also determined in 6 out of the 55 studied ones. The closest negative relations were for the fruit ratio vs. fruit width and fruit length vs. calyx ratios.

**Table 5:** Linear correlation coefficient (r) and its significance between the quantitative traits used for phenotyping in the collection of the 31 eggplant landraces cultivated in Spain.

**Trait**	**Fr Length**	**Fr Weight**	**Fr Width**	**Lf Length**	**Lf Width**	**N° calix pickels**	**N° Fl**	**P Length**	**P Width**	**Fr L/W**
Calyx L/Fr L	−0.6542***	0.0154	0.262	0.0743	0.0846	0.3839*	0.3658*	**–**0.2841	**–**0.0477	−0.557**
Fr Length		0.124	**–**0.3517	0.1206	**–**0.0733	**–**0.0277	**–**0.1463	0.4281*	0.0742	0.7692***
Fr Weight			0.858***	0.4650**	0.4088*	0.4721**	**–**0.1219	**–**0.2166	0.4282*	−0.4887**
Fr Width				0.3590*	0.3974*	0.4208*	**–**0.0322	−0.4110*	0.3886*	−0.8218***
Lf Length					0.7177***	0.0893	0.2532	**–**0.0024	0.248	**–**0.1921
Lf Width						0.2524	0.1997	−0.3580*	0.2219	**–**0.2662
N° calix pickels							0.0342	**–**0.3069	0.1363	−0.311
N° Fl								**–**0.0549	**–**0.0874	**–**0.1027
P Length									0.3004	0.4856**
P Width										**–**0.1965

***, **, * indicate significance at p < 0.001, p < 0.01, p < 0.05 for r. P, plant; Lf, leaf; Fl, flower; Fr, fruit; L/W, length/width ratio. These significant values have been highlighted in bold.

### Nutraceutical Characteristics

The fruit of three eggplant landraces (B14, B16, B17) were characterised to establish fruit quality. Significant differences (*p* < 0.05 or *p* < 0.001) were found among the average values in the selected eggplant landraces for all the analysed nutraceutical compounds, but not in the DW percentage (Table 6).

**Table 6:** Fruit quality traits in three local eggplant landraces cultivated in Spain.

**Trait**	**B14**	**B16**	**B17**	
DW (%)	23.09 ± 4.60	21.87 ± 3.76	24.38 ± 8.53	ns
Pulp L*	72.74 ± 87.11^b^	73.22–77.23 ±^b^	71.27 ± 88.48^a^	**
Pulp a*	−5.57±−3.72^b^	−1.14±−1.05^a^	−2.27±−0.61^a^	***
Pulp b*	17.23 ± 22.87^a^	9.57 ± 10.62^b^	7.76 ± 12.27^b^	***
DPPH (%)	38.29 ± 11.03^a^	19.75 ± 6.74^b^	21.71 ± 3.89^b^	***
Phe (*mg g^−1^FW*)	4.47 ± 1.21^a^	2.53 ± 0.64^b^	2.61 ± 0.65^b^	***
Flav (*mg 100 g^−1^FW*)	65.7 ± 23.90^a^	27.25 ± 11.64^b^	26.10 ± 8.99^b^	***
Asc (*mg 100 g^−1^FW*)	10.95 ± 3.67^a^	4.82 ± 1.10^b^	12.75 ± 3.46^a^	***
Car (*μg g^−1^FW*)	3.88 ± 0.17^a^	1.65 ± 0.21^b^	1.78 ± 0.16^b^	***
Sugars (*g 100 g^−1^FW*)	5.34 ± 1.21^b^	4.84 ± 0.28^b^	6.48 ± 0.96^a^	*

Values are the mean ± SD of n = 5 for dry weight and sugars, n = 20 for colour parameters and n = 10 for antioxidant traits. Different letters in a row indicate significant differences at p < 0.05 (LSD test). ***, **, * and ns denotes significance at p < 0.001, p < 0.01, p < 0.05 and non-significant values, respectively. DW, Dry Weight; L*, lightness value; a*, greenness/redness value; b*, blueness/yellowness value; DPPH, Antioxidant capacity; Phe, Phenols; Flav, Flavonoids; Asc, Ascorbic acid; Car, Carotenoid concentration.

#### Fruit DW Percentage

The fruit DW (Table 6) range was 11.57–42.64% for the studied landraces. The mean values for cultivars B14, B16, and B17 were 24.37, 21.87, and 23.09%, respectively, and no significant differences were observed among landraces.

#### Pulp Colour

The L^*^, a^*^, b^*^ colour parameters, measured on the fruit inner pulp after cut, (Table 6) ranged from 71.27 to 88.48 (L^*^), −2.38 to 1.6 (a^*^) and 15.11–22.87 (b^*^) for the studied landraces. Focusing on colour parameter L^*^, significant differences were found between landraces, turning B17 to own a lighter pulp colour (1.07-fold higher value). When analysing colour parameter a^*^, it was observed that landrace B14 had a greener pulp tonality (3.62-fold higher value). Also, in accordance with b^*^ parameter data, landrace B14, showed a much more yellowish pulp (1.97-fold higher value), what differentiated this variety from B16 and B17.

#### Nutraceutical Compounds and Antioxidant Capacity

##### Antioxidant Capacity

Antioxidant capacity was determined by the DPPH assay (Table 6) and its range was 9.97–62.65%. Landrace B14 had a statistically higher antioxidant capacity (mean value of 38.29 mg g^−1^ FW) compared to B16 and B17, with no differences found between them. Antioxidant capacity was 17.56% higher (1.8-fold) in B14.

##### Phenols

The total phenolic content (Table 6) for the three different eggplant cultivars ranged from 1.56 to 7.48 mg g^−1^ FW. B14 obtained a significantly higher mean value for phenolic content (4.47 mg g^−1^ FW), with no differences between B16 and B17 (2.53 and 2.61 mg g^−1^ FW, respectively). Phenolic concentration was 1.7-fold higher in landrace B14, which was 42% higher than for the other varieties.

##### Flavonoids

The total flavonoid content (Table 6) ranged between 5.75 and 118.63 mg 100 g^−1^ FW among the three landraces. Significant differences were found in the three landraces in relation to the total flavonoid content. Landraces B16 and B17 did not show any significant differences in the flavonoid concentration, and their mean values were 27.25 and 26.1 mg 100 g^−1^ FW, respectively. B14 stood out for its high flavonoid content (mean value of 65.7 mg 100 g^−1^ FW). The flavonoid concentration was 59.4% higher in B14, which was 2.5-fold higher than B16 and B17.

##### Ascorbic Acid

The ascorbic acid content (Table 6) range was 3.45–18.45 mg 100 g^−1^ FW for the three landraces. Significant differences were found in the three landraces. Landrace B16 had a statistically lower ascorbic acid content (mean value of 4.82 mg 100 g^−1^ FW) compared to B14 and B17 (mean values of 10.94 and 12.75 mg 100 g^−1^ FW, respectively), with not differences between them. The ascorbic acid concentration was 60% lower in B16, which is 2.5-fold lower than B14 and B17.

##### Carotenoids

The total carotenoid content (Table 6) range was 1.46–4.06 μg g^−1^ FW for the three landraces. The ANOVA analysis showed that landrace B14 had the highest carotenoid content (mean value of 3.88 μg g^−1^ FW). Accessions B16 and B17 did not show significant differences between them and, respectively, presented 1.65 and 1.78 μg g^−1^ of FW carotenoids as the mean value. The carotenoid concentration was 55.6% (2.27-fold) lower in B16 and B17 compared to B14.

#### Soluble Sugars

The sugar content (Table 6) for the different eggplant cultivars ranged from 4.85 to 7.62 g 100 g^−1^ FW, which is a 1.57-fold increase in content. Considerable differences were found in the studied landraces. B17 had a significantly higher mean value for sugar content (6.48 g 100 g^−1^ FW), and no differences were reported between B14 and B16 (5.336 and 4.96 g 100 g^−1^ FW, respectively). Sugar content was 20.64% higher in B17, which is 1.25-fold increase compared to B14 and B16.

#### Correlation Between Antioxidant Compounds

In order to estimate the contribution of the quality traits in the pulp of the fruits, several correlation analyses were carried between the different combinations of the percentage of DW, colour, nutraceutical compounds and sugar data (Table 7). The pairwise coefficients showed a positive correlation and a statistical significance for 11 pairs and only two negative correlations. While DW was not correlated with any trait, the colour parameters in the pulp showed marked correlations. The strongest and most positive ones were registered between b^*^ value and four of the five nutraceutical compounds (*r* = 0.9604 for carotenoids and *r* = 0.5769–0.6327 for DPPH, phenols, and flavonoids). By contrast, a strong but negative correlation was observed between a^*^ and carotenoids content (*r* = −0.9771) while it was moderate and positive between L^*^ and sugar content (*r* = 0.6226). When comparing nutraceutical compounds, four strong significant and positive correlations were recorded between the combinations of DPPH vs. phenolics, DPPH vs. flavonoids, DPPH vs. carotenoids, and phenolics and flavonoids, where the coefficient r ranged from 0.7955 to 0.8322. Phenols vs. carotenoidsalso showed moderate and positive correlation (*r* = 0.6898). Related to sugar content, it was positively correlated with carotenoids content (*r* = 0.6302).

**Table 7:** Linear correlation coefficient (r) and its significance between fruit quality traits (dry weight, pulp colour, nutraceutical compounds, and sugars) in the collection of the three eggplant landraces (B14, B16, B17) cultivated in Spain.

**Trait**	Pulp L*	Pulp a*	Pulp b*	**DPPH**	**Phe**	**Flav**	**Asc**	**Car**	**Sugars**
% DW	−0.2284	0.007	−0.03	−0.0694	−0.0077	−0.0203	−0.0677	−0.0969	−0.1582
Pulp L*		−0.2432	−0.3297*	−0.136	−0.145	−0.1223	0.2949	0.2295	0.6226*
Pulp a*			0.0245	−0.0267	0.0202	−0.0087	−0.3182	−0.9771***	0.088
Pulp b*				0.6327***	0.5864***	0.5769***	0.3631	0.9604***	−0.1974
DPPH					0.8263***	0.8322***	0.1716	0.7955**	−0.2803
Phe						0.8256***	0.1461	0.6898*	−0.3188
Flav							0.1289	0.4348	−0.3722
Asc								0.5248	0.6302*
Car									0.2121

***, **, * indicate significant at p < 0.001, p < 0.01, p < 0.05 values for r. DW, Dry Weight; L*, lightness value; a*, greenness/redness value; b^*^, blueness/yellowness value; DPPH, Antioxidant capacity; Phe, Phenols; Flav, Flavonoids; Asc, Ascorbic acid; Car < Carotenoid concentration. These significant values have been highlighted in bold.

## Discussion

The morphological diversity of eggplant landraces has been the subject of many studies (Furini and Wunder, 2004; Prohens et al., 2005; Behera et al., 2006; Muñoz-Falcón et al., 2008, 2009; Özer et al., 2011; Kaushik et al., 2016). These surveys are necessary since they provide germplasm banks with very useful information, and they contribute to optimise plant breeding programmes. According to Uddin et al. (2015), clustering accessions in different groups may be useful for providing a basis for further crop improvement. Many characterisation studies based on standardised morphological and agronomic descriptors developed by the International Board for Plant Genetic Resources have been performed in eggplants, and have demonstrated that they are suitable for providing very helpful information for eggplant breeders (Prohens et al., 2005; Muñoz-Falcón et al., 2009; Boyaci et al., 2015). In view of the success of these surveys, the characterisation of the selected valencian varieties was made following IBPGR guidelines. Furthermore, nutraceutical quality also defines a relevant role in crop improvement (Jenks and Bebeli, 2011), mainly due to eggplants' antioxidant content (Peschel et al., 2006), including polyphenols, ascorbic acid and carotenoids (Du et al., 2009), among others.

The PCA has been previously used to determine the most important traits for landrace characterisation of different species, such as sweet potato (Yada et al., 2010), spider plant (Wasonga et al., 2015), African tomato landraces (Tembe et al., 2018) and eggplant (Cericola et al., 2013; Uddin et al., 2015; Tembe et al., 2020). According to our results, when subjecting the phenotypic data of the 31 landraces to the PCA analysis, nine principal components were established and corresponded to an 80% total variation. Of the nine components, none explained more than 25% of the diversity among landraces. For this reason, from the PCA analysis we inferred a wide diversity among accessions, even if landraces belonged to the same Mediterranean area. Muñoz-Falcón et al. (2008) suggested that local conditions, in addition to the selection processes followed by farmers, generated a differentiation in the eggplants of the same origin. Likewise, together with this diversification process, as eggplants are generally self-pollinated plants (Pessarakli et al., 2004), the genetic isolation of various eggplant populations may has been favoured. The mayor principal component that explained 23.3% of the total variability correlated mainly with the fruit descriptors. This separation of accessions associated with fruit traits has also been described by other authors (Prohens et al., 2005; Özer et al., 2011; Tembe et al., 2020), which confirms that the morphological variation in the organ for which a crop is selected widens during the domestication process (Meyer and Purugganan, 2013). Despite the genetic bottleneck that eggplant domestication has undergone (Lester and Hasan, 1991), considerable diversity is found among landraces, unlike that seen in commercial varieties, especially in F_1_ hybrids (Muñoz-Falcón et al., 2009). Although commercial hybrids have been selected for traits like earliness, yield, lack of prickles or colour, the diversity of other morphological characters has been narrowed (Prohens et al., 2005).

The correlation analysis measures the degree of relation between the selected phenotypic quantitative traits, distinguishing remarkable characters for crop improvement (Kranthi and Celine, 2013; Kumar et al., 2016). Of the obtained values, two clear trends were observed. The higher the plant height value is, the longer and lighter fruits are, while the wider the plant is, the wider and heavier its fruits. Leaf length and width were positively and significantly correlated with the average fruit weight (*r* = 0.4650 and *r* = 0.4088) and width (*r* = 0.359 and *r* = 0.3974). A larger foliar area can offer better accumulation of photosynthates in plants, to ultimately produce heavier and larger fruits (Kumar et al., 2016). A statistically significant relation existed between the number of calyx prickles with fruit weight (*r* = 0.4721) and width (*r* = 0.4208), while the absence of calyx prickles is desirable for harvest processes or consumer handling. On the contrary, a negative, but statistically significant, correlation occurred between the calyx length ratio and fruit length (*r* = −0.6542). Owning a short calyx (~20%) is a desirable attribute from the phytosanitary point of view since it helps to prevent eggplants from white mites, Botrytis cinerea and several fungal diseases, whose presence is favoured by relative high humidity as petals are adhered between the calyx and fruit (Aramendiz et al., 2006). On this matter, and in relation to this trait, slightly heavy and elongated fruits would be preferable. Flower number per inflorescence was positively correlated with the calyx length ratio (*r* = 0.3658), while showing a negative tendency toward a relation with big and heavy fruit. Altogether would mean that plants with great number of flowers, which are also able to develop many eggplants per plant, would develop small calibre fruits. This tendency has also been described by other authors in eggplants (Cericola et al., 2013; Tembe et al., 2020). In general terms, pubescence on leaves, lack of prickles on leaves and the calyx, erect growth habit and long fruit development are desirable attributes that facilitate the agronomic work of eggplant crops, especially concerning the harvest. They also meet the needs of both producers and final consumers (Aramendiz et al., 2006).

Regarding fruit pulp quality, our study established significant differences in landraces B14, B16, and B17 as the antioxidant capacity of B14 was 17.56% higher than in the other cultivars. Other authors have also reported wide variability in antioxidant capacity in eggplant landraces (Chumyam et al., 2013; Kaur et al., 2014; Niño-Medina et al., 2014; Sukprasansap et al., 2019). Total antioxidants levels have been widely established in eggplant, but very little attention has been paid to their distribution within the fruit and their stability in different genotypes (Stommel and Whitaker, 2003). To our knowledge, very few studies claim that the major antioxidant capacity is found in fruit pulp (Jung et al., 2011). Likewise from the information obtained from this minority of studies, it is known that the inner or central part of pulp has the greatest antioxidant capacity (Zaro et al., 2014a), for this reason our results could seem exaggerated compared to studies which have worked with whole fruits.

Among vegetables, eggplants are an important source of phenolics, flavonoids and ascorbic acid, all of which are powerful antioxidants (Vinson et al., 1998). Phenolics in eggplant have been identified as the major bioactive compounds responsible for their antioxidant effects (Kwon et al., 2008) and genotypes are highly diverse in the proportions of these compounds measured by spectrophotometry (Plazas et al., 2013). This statement agrees with the results herein obtained, where landrace B14 stood out for its high phenolic content (4.47 mg g^−1^ FW), which was 42% higher than in B16 (2.53 mg g^−1^ FW) and in B17 (2.61 mg g^−1^ FW). These results are higher than the values reported by several authors (Ninfali et al., 2005; Hanson et al., 2006; Raigón et al., 2008; San José et al., 2013; Kaur et al., 2014), but are similar to those obtained by Plazas et al. (2013) and Niño-Medina et al. (2014). Also, when comparing these eggplants with other crops, we found that eggplant showed higher values than most of the vegetables and just few species as green pepper (2.47 mg g^−1^ FW) (Ribarova et al., 2005), spinach (2.69 mg g^−1^ FW) and red onion (2.53–3.11 mg g^−1^ FW) reached similar levels. Moreover, even if the phenolic content in eggplant is comparable to that found in many types of fruits, such as in strawberry (3.64 mg g^−1^ FW) (Lin and Tang, 2007), plum (3.04 mg g^−1^ FW), blueberry (4.25 mg g^−1^ FW), and blackberry (2.47 mg g^−1^ FW) (Jab ł oń ska-R yś et al., 2009), significantly exceeds the phenolic content of many others as apples (around 1 mg g^−1^ FW), sweet cherries (7.88 mg g^−1^ FW), raspberries (1.79 mg g^−1^ FW) or black grapes (2.13 mg g^−1^ FW) (Ribarova et al., 2005).

Significant differences were detected when determining the total flavonoid concentration of the three selected landraces. The flavonoid concentration was 59% higher in landrace B14 (65.7 mg 100 g^−1^ FW), while it came close to 26 mg 100 g^−1^ FW in landraces B16 and B17. However, other studies performed in different eggplant landraces unseat our candidates because they had much higher flavonoid concentrations; 1.733 mg 100 g^−1^ FW (Bor et al., 2006), 1.991–3.954 mg 100 g^−1^ FW (Akanitapichat et al., 2010), 370 mg 100 g^−1^ FW (Nayanathara et al., 2016), 142.16–718 mg 100 g^−1^ FW (Koley et al., 2019), 152.4–392 mg 100 g^−1^ FW (Nwanna et al., 2019). Nevertheless, other authors have presented comparable results to ours (Ninfali et al., 2005; Frond et al., 2019; Dong et al., 2020), and even much lower ones (Boulekbache-Makhlouf et al., 2013; Kaur et al., 2014; Zambrano-Moreno et al., 2015). All together, these works suggest a very wide diversity among eggplant landraces and/or crop management, climate conditions, etc. Although the obtained results on our varieties for flavonoids were not striking, it must be remembered that the key lies in the wide variability among traditional varieties, and in the need to account for several bioactive compounds to determine the whole antioxidant capacity of a given variety. Likewise, the present results are comparable to those obtained in other vegetables like beetroot (62.8 mg 100 g^−1^ FW), red onion (36–56 mg 100 g^−1^ FW) (Lin and Tang, 2007) and carrot (26.7 mg 100 g^−1^ FW) (Ribarova et al., 2005), but higher than those expected in pepper (7–11 mg 100 g^−1^ FW)(Lin and Tang, 2007) or tomato (4–26 mg 100 g^−1^ FW) (Slimestad et al., 2008). However, the flavonoid content of eggplant is much lower compared to that of leafy vegetables, for example, spinach (133.1 mg 100 g^−1^ FW) (Lin and Tang, 2007) and lettuce (97.2 mg 100 g^−1^ FW) (Ribarova et al., 2005). When making this same comparison with different fruits, we observed that the amount of flavonoids found in most of them falls within the range of our results, with some exceptions; plum (136.2 mg 100 g^−1^ FW), dogwood berry (91.4 mg 100 g^−1^ FW) (Ribarova et al., 2005), and mulberry (250.1 mg 100 g^−1^ FW) (Lin and Tang, 2007).

Other non-destructive methods, using remote optical methods like hyperspectral analysis and multispectral fluorescence, are also suitable for predicting the right content of some polyphenols with a good correlation (Sytar et al., 2017). Therefore, non-invasive methods enable a rapid pre-screening (positive or negative) of tens or several hundred thousands of individuals from which the best samples will be selected and tested by metabolomic analyses, which will greatly increase the efficiency of the entire process (Sytar et al., 2020) and allow monitoring the evolution of these compounds along the growth cycle. However, the use of these techniques still needs to be perfected and facilitated.

The significant differences found across accession in vitamin C content suggest genotype dependence for this trait in eggplant. The average ascorbic acid concentration in the different landraces ranged from 3.46 to 18.47 mg 100 g^−1^ FW. These values are consistent with several studies carried out on eggplant landraces (Bidaramali et al., 2020; Quamruzzaman et al., 2020). Some studies, such as Prohens et al. (2014) have even found major differences between traditional varieties and commercial hybrids, meaning that, on average, landraces present higher ascorbic acid content. Although many foods contain a similar vitamin C content, such as onion, pineapple (Szeto et al., 2002), blackthorn (Jabłońska-Ryś et al., 2009) and apple (Kapur et al., 2012), the ascorbic content of many other popular fruit and vegetables, such as orange, kiwi, grapefruit, strawberry (Szeto et al., 2002), blueberry (Jabłońska-Ryś et al., 2009), pepper and date (Kapur et al., 2012), is much higher than that found in eggplants. Ascorbic acid is a potent antioxidant, so the relatively low ascorbic acid content in eggplant fruits may limit the whole plant's antioxidant capacity (Hanson et al., 2006). Nevertheless, this deficiency may be balanced out with its high phenolic content.

The antioxidant activity of carotenoids and their biochemical properties that influence disease prevention have also been discussed (Stahl and Sies, 2005). The average total carotenoid concentration in the eggplant landraces ranged from 1.46 to 4.06 μg g^−1^ FW, which gave a mean value of 2.5 μg g^−1^ FW. Very few studies have been carried out on eggplants in which total carotenoids content ranged from 0.44 to 1.22 μg g^−1^ FW (Mangels et al., 1993) or obtained 1.32 μg g^−1^ FW as the mean value (Qudah and El-Qudah, 2009). According to the National Nutrient Databases for Standards Reference (USDA), the mean carotenoid content in eggplants is 0.16 μg g^−1^ FW. Although, the local varieties herein used appear to have a much higher carotenoid content on average, in other crops as in zucchinis (28.34 μg g^−1^ FW) (Qudah and El-Qudah, 2009), and green peppers (20 μg g^−1^ FW) (Gisbert-Mullor et al., 2020). These concentrations are more than eight times as much compared to the carotenoid content in eggplant, but are almost worthless compared to red peppers (130 μg g^−1^ FW) (Gisbert-Mullor et al., 2020) and carrots (95.93 μg g^−1^ FW) (Qudah and El-Qudah, 2009).

The correlations found in this study between antioxidants, fall in line with those presented by Barreto et al. (2009), Ramaiya et al. (2013), and Fratianni et al. (2020), who suggested that antioxidant capacity was positively linked with the amount of total polyphenols, and was less related to the content of both carotenes and ascorbic acid. In this study, statistically remarkable relationships were found between phenolics and carotenoids (*r* = 0.7955), but not between ascorbic acid and phenols. Andarwulan et al. (2012) stated that ascorbic acid is known to contribute to total phenolic content, even when no correlation is observed between them. A positive relationship between these two parameters was detected by Hanson et al. (2004) in tomato, which supports this idea. Finally, a moderate and statistically significant correlation was observed between ascorbic acid and sugar content (*r* = 0.6302). This same relationship was detected by Ramaiya et al. (2013) in papaya, associating it to the common and complex interactions existing between organic acids and sugars.

Some correlations between nutraceuticals and colorimetric parameters in the pulp were also registered in the experiment. A moderate correlation was detected between the L^*^ parameter and the sugar content of the pulp, a relationship also highlighted by Orak (2007) in red grapes pulp. It is known that eggplant fruits accumulate sugars preferentially in the inner pulp (Zaro et al., 2014b). Therefore, in our case, as the colorimetric data was taken in this particular region ofthe pulp, the concentration of sugars may have modified the value of L^*^. In addition, parameter b^*^ was related to antioxidants as a general rule, a fact also mentioned by Orak (2007). Finally, a^*^ appears to be negatively related to carotenoids, which is to be expected since this parameter detects reddish shades and carotenoids are known to provide from yellowish to reddish tones (Wibowo et al., 2015).

Health-conscious consumers generally focus on the antioxidant capacity, and the phenolic and vitamin contents of foods (Gürbüz et al., 2018). However, fruit quality is determined primarily by taste, and a major component of taste is sugar content (Burger et al., 2006). Hence, its analysis is recommended in eggplant, as fruits are believed to be rich in this compound and could therefore satisfy consumers (Best et al., 2010). Even though significant differences were found among the three landraces analysed in this assay, and landrace B17 stood out for its high sugar content, compared to data from other studies on eggplants (Passam and Karapanos, 2008; Hernández-Hernández et al., 2011; San José et al., 2013; Zaro et al., 2014b; Pohl et al., 2019; Bidaramali et al., 2020), it is observed that sugar concentration is almost doubled in our landraces.

## Conclusions

The herein reported results showed the high degree of diversity among the selected traditional eggplant varieties. Among morphological characteristics that may be of interest for handling jobs like crop harvesting, are included having an erect growth habit, low branch density, lack of hairiness on leaves, no prickles on the calyx and the development of elongated and not excessively heavy fruits. Between groups G1 and G2, which include similar varieties to those marketed today, landraces B4, B12, and B19 could be highlighted based on the previous traits. Trade in the G3 and G4 varieties could also be promoted because: white-fruited varieties (G3) produce many flowers and somewhat elongated fruits of an attractive colour for consumers, while the reddish fruit entries (G4) produce elongated but not too heavy fruits, with a few thorns on the calyx, which could be interesting options. As the nutritional profile is helpful for promoting the commercialisation and consumption of local varieties, and according to the nutritional quality part of this study, variety B14 could be promising for human consumption, mainly for its antioxidant properties. Taken together, this information could be relevant for future plant breeding programmes to obtain easily manageable and harvestable eggplant varieties.

## Data Availability Statement

The original contributions presented in the study are included in the article/Supplementary Material, further inquiries can be directed to the corresponding author/s.

## Author Contributions

ÁC, M-RM-C, and EM-I: conceptualization, validation, and investigation. ÁC, EM-I, M-RM-C, JM, and RM-F: methodology. EM-I and M-RM-C: formal analysis and writing—original draught preparation. ÁC and M-RM-C: data curation. ÁC, SS, MD, and JV: resources, writing—review and editing, supervision, and funding acquisition. All authors have read and agreed to the published version of the manuscript.

## Conflict of Interest

The authors declare that the research was conducted in the absence of any commercial or financial relationships that could be construed as a potential conflict of interest.
